# Revising BMI Cut-Off Points for Overweight and Obesity in Male Athletes: An Analysis Based on Multivariable Model-Building

**DOI:** 10.3390/nu17050908

**Published:** 2025-03-05

**Authors:** Chiara Milanese, Leila Itani, Valentina Cavedon, Dana Saadeddine, Silvia Raggi, Elisa Berri, Marwan El Ghoch

**Affiliations:** 1 Department of Neurosciences, Biomedicine and Movement Sciences, University of Verona, 37129 Verona, Italy; chiara.milanese@univr.it (C.M.); valentina.cavedon@univr.it (V.C.); 2 Department of Nutrition and Dietetics, Faculty of Health Sciences, Beirut Arab University, Riad El Solh, Beirut P.O. Box 11-5020, Lebanon; l.itani@bau.edu.lb; 3 Center for the Study of Metabolism, Body Composition and Lifestyle, Department of Biomedical, Metabolic and Neural Sciences, University of Modena and Reggio Emilia, 41125 Modena, Italy; dana.saadeddine@unimore.it; 4 Degree Course of Dietetics, Innovation and Research Training Service, Azienda Ospedaliero-Universitaria di Modena, 41124 Modena, Italy; silvia.raggi@unimore.it (S.R.); elisa.berri@unimore.it (E.B.); 5 Department of Primary Care, Azienda Unità Sanitaria Locale-IRCCS di Reggio Emilia, 42123 Reggio Emilia, Italy

**Keywords:** athletes, BF, body composition, cut-off, sport, overweight, obesity

## Abstract

Background: Body composition in athletes is characterized by pronounced muscle mass and low body fat (BF). Over and excessive adiposity are thus expected in athletes at higher body mass index (BMI) levels than those suggested by the World Health Organization (WHO). Therefore, we aimed to test the validity of WHO BMI cut-off points for overweight and obesity, respectively (i.e., ≥25 kg/m^2^ and 30 kg/m^2^) in young male athletes from different sport disciplines in Italy. Methods: This study includes 622 male young adult athletes of mean age 25.7 ± 4.7 years who were initially categorized according to the WHO BMI classification, and then re-categorized by adiposity status based on total BF% as measured by dual-energy X-ray absorptiometry (DXA). A predictive equation has been developed utilizing multivariable model-building to predict the best BMI cut-offs for identifying overweight and obesity in this population. The agreement between the different classification systems was assessed with the kappa statistic (κ). Results: According to the WHO BMI classification, 451 (72.5%) individuals were of normal weight, 148 (23.8%) were with overweight and 23 (3.7%) were with obesity, but based on the total BF%, 598 (96.1%) were of normal weight, and only 19 (3.1%) were with overweight and 5 (0.8%) were with obesity, revealing a weak agreement between the two classification systems (WHO BMI vs. BF%; κ = 0.169). On the other hand, new BMI cut-off points were identified (BMI ≥ 28.2 kg/m^2^ for overweight and 33.7 kg/m^2^ for obesity) and showed good agreement with the BF% classification system (κ = 0.522). Conclusions: The currently used WHO BMI cut-offs are not suitable for determining weight status in young male athletes, and since the newly proposed ones demonstrated a good performance, these should be implemented in new guidelines.

## 1. Introduction

The Quetelet index, identified as the body mass index (BMI), is calculated as an individual’s bodyweight, usually expressed in kilograms, divided by their height in meters squared [1,2]. The BMI remains the most used surrogate measure of adiposity at both clinical and epidemiological levels [3,4,5,6]. When classifying overweight or obesity in adults, the World Health Organization (WHO) relies on BMI cut-off points of ≥25 and 30 kg/m^2^ in Caucasians to indicate overweight and obesity, respectively, in almost all age and gender groups, including the athletic population [7].

In this context, and because athletes’ body composition is usually characterized by pronounced muscle mass and low body fat (BF) [8,9,10,11], it should be expected that over and excessive adiposity (i.e., overweight or obesity) may occur at higher body mass index (BMI) cut-off points than those proposed by the WHO [12]. In fact, early studies conducted among male athletes (i.e., runners and handball players) showed that BMI is not a good predictor of adiposity in this population, as it can only be indicative of body size [13]. In addition, within the athletic population, athletes that appear to have a similar body mass (i.e., body weight, BMI) seem to have significantly different levels of body fat percentage (BF%), a finding that has been attributed to the sport disciplines they regularly practice [14].

More specifically, the WHO BMI cut-offs of 25 and 30 kg/m^2^ may overestimate over and excessive adiposity (i.e., obesity) in athletes, as the classification of adiposity status based on BF remains the most accurate method [15]. For instance, in one study conducted on male athletes who played American football, almost a quarter of the sample was considered overweight when relying on traditional WHO BMI cut-off points (i.e., ≥25 kg/m^2^). However, after assessing BF% by means of dual-energy X-ray absorptiometry (DXA), many of those were found to have a low BF%, not reaching values that could be categorized as being with overweight or obesity [16]. Despite all the above-mentioned considerations, when classifying weight status in sportsmen and sportswomen, many sport societies and organizing committees still rely on the WHO BMI classification system [7].

In this direction, it has been demonstrated and is now widely accepted that BMI has several limitations. In particular, it is unable to discriminate between body composition compartments, namely fat and lean mass (LM) [17], making it unsuitable for use among athletes, who are characterized by an increased muscularity and decreased adiposity in comparison to non-athlete individuals. The universal BMI cut-off points of 25 and 30 kg/m^2^ for overweight and obesity, respectively, are therefore inappropriate and debatable, as has been highlighted in the literature [18,19]. In fact, several studies have already showed that a higher BMI does not necessarily represent over and excessive adiposity in several athletic populations [20,21]. However, very few works had the primary aim of establishing suitable BMI cut-offs to better identify over and excessive adiposity status in this specific population (i.e., athletes) [22].

Therefore, our study accordingly had the primary aim of assessing to what extent the WHO BMI cut-off points for overweight and obesity classification (i.e., 25 and 30 kg/m^2^, respectively) are accurate for usage as an indicator of over and excessive adiposity in a population composed of young Italian adult male athletes. In case they were found to be not accurate, this research aimed to identify new BMI cut-offs that are more suitable for use wherever this is necessary.

## 2. Materials and Methods

### 2.1. Participants and Design of the Study

Our study has a cross-sectional observational design. The participants were referred to and subsequently enrolled in the Department of Neurosciences, Biomedicine and Movement Sciences, University of Verona, Verona, Italy, between June 2019 and May 2023. Individuals met the eligibility criteria for participation if they were male, were athletes officially practicing a sport discipline at a competitive level (i.e., city/province or region/district or at a national or international level) and had undergone a total body composition assessment by means of a dual-energy X-ray absorptiometry (DXA) scan. A total of 1240 participants were found to be eligible and were then checked against the inclusion and exclusion criteria. The inclusion criteria involved having (i) an age ≥ 20 years and (ii) a BMI ≥ 20 kg/m^2^, as well as (iii) a white Caucasian ethnicity. Participants were excluded if they were (i) female or had (ii) an age < 20 years, (iii) a BMI < 20 kg/m^2^ (iv) or a different ethnicity (i.e., Hispanic, black or Asian). A total of 622 male individuals with different body weight statuses according to the WHO BMI classification, of normal weight (n = 451), overweight (n = 148) or obesity (n = 23), were included.

This study was conducted according to the guidelines of the Declaration of Helsinki and approved on 13 November 2019 by the Institutional Review Board of the University of Verona (No. #26/2019). All participants’ personal data were treated according to European/Italian privacy laws, and informed written consent was obtained.

### 2.2. Body Weight and Height

Body weight and height were measured at the nearest 0.1 kg and 0.01 m with an electronic scale (Tanita electronic scale BWB-800 MA, Wunder SA.BI. Srl, Milano, Italy) and Harpenden stadiometer (Holtain Ltd., Crymych, Pembs, UK), respectively [23]. These measures were performed with the participant wearing light clothes and their shoes removed. The BMI was calculated according to the standard formula as the ratio of body weight expressed in kilograms divided by the square of the height in meters.

### 2.3. Body Composition

Body composition was determined by DXA scan (QDR Explorer W; Hologic, Bedford, MA, USA; fan-beam technology, software for Windows XP version 12.6.1) based on the procedure provided by the manufacturer [24]. In order to ensure consistent measurements, the DXA scan was run on a daily basis using a standard anthropomorphic spine phantom from the manufacturer, and the same operator carried out the DXA measurements of all the participants included in the study. When the scans were performed, no special preparations were made except for giving clear instructions to participants to keep only their underwear on and to remove any metal accessories during the testing procedure [25]. The entire sample included in the research was categorized according to age- and gender-specific overweight and obesity BF% cut-off points, as mentioned below [26]:

- BF% ≥ 21% for overweight;
- BF% ≥ 26% for obesity.

### 2.4. Statistical Analysis

To estimate the new cut-off points, multivariable model-building was utilized to calculate a predictive equation for BMI using BF%. To satisfy the assumption of the models for BMI normality across BF% and the normality of standardized residuals, Ln BMI was employed in the model [27]. The best model was selected based on R^2^. The procedure was applied after the non-linearity of the association between BMI and BF% was confirmed by a cumulative sum (CUSUM) test [28]. The model was adopted to compute the predicted BMI (pBMI) in the sample by calculating the antilog from the new model. To confirm the ability of the derived model to predict BMI correctly, Pearson’s correlation coefficient was determined between the measured BMI and pBMI values, and a regression analysis was carried out by regressing the measured BMI on the pBMI. Obtaining a regression line with a slope of 1 and an intercept of 0 indicated accurate prediction with no bias. A regression line with a slope significantly deviating from 1 suggested that a unit change in pBMI did not correspond to one in measured BMI. A *t*-test was performed to confirm the slope was not different from 1 with the “test” function in Stata (version 15) [29], as well as a paired *t*-test to ascertain that the difference between the measured BMI and pBMI did not deviate from 0. A CUSUM test was also conducted to confirm a linear association between predicted and measured BMI.

To derive the new BMI cut-off points, the BF% cut-off points for overweight (21%) and obesity (26%) [26] were substituted in the derived model, then the antilog was calculated to obtain the new BMI cut-off points. The chi-squared test for independence was used to compare the distribution of BMI categories across categories of BF%. The kappa statistic was utilized to assess the agreement of the WHO classification and the proposed BMI with BF% categories. A kappa value ≤ 0 is considered poor, 0.01–0.20 is slight, 0.21–0.40 is fair, 0.41–0.60 is moderate, 0.61–0.80 is substantial and 0.81–1 is almost perfect [30]. The descriptive analysis is presented as means ± SD for continuous variables and frequencies and proportions for categorical variables. All tests were considered significant at *p* < 0.05. All statistical analysis was performed with SPSS (version 27) [31], NCSS (version 24.0.3) [32] and Stata (version 15) [29].

## 3. Results

The anthropometric characteristics of the study participants are shown in Table 1. A total of 622 male athletes were included with a mean age of 25.7 ± 4.7 years and a mean BMI of 24.2 ± 2.6 kg/m^2^ that is closer to the normal BMI. The mean total LM was 65.8 ± 8.0 kg, constituting 82.8 ± 3.2% of the total body weight. The mean total BF was 10.6 ± 4.1 kg, forming 13.1 ± 3.4% of the total body weight. According to the BF classification system, 598 (96.1%) had a normal body fat composition, 19 (3.1%) were with overweight and 5 (0.8%) were with obesity.

Table 2 presents the agreement analysis between BF classification [26] for normal weight, overweight and obesity and the WHO BMI cut-off points [7]. According to the WHO classification, in total, 451 (72.5%) were classified as having a normal weight and 171 (27.5%) with overweight or obesity. The WHO cut-off points misclassified those with normal BF by 24.9% as being with overweight or obesity, while it captured 91.7% of those with overweight or obesity according to BF. The agreement analysis reflected a poor agreement between the two classifications (kappa = 0.169).

**Figure 1:**
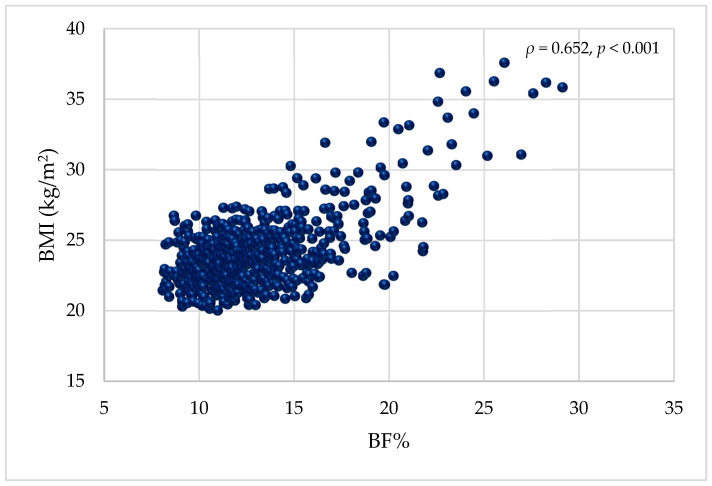
Scatter plot illustrating the association between BF% and BMI (kg/m^2^). BMI = body mass index; BF% = body fat percentage.

The scatter plot in Figure 1 illustrates the correlation between body fat percentage as a measure of obesity and BMI (kg/m^2^). A significant positive correlation of ρ = 0.652 (*p* < 0.001) was observed, reflecting the ability of body fat percentage to predict BMI. Results from the CUSUM test for linearity did not support a linearity assumption reflecting a non-linear association (*p* < 0.05) between BMI and BF%.

Using multivariable model-building by regressing Ln BMI on BF% revealed a quadratic association of the form:

(1) $$\mathrm{Ln}\;\mathrm{BMI}=3.32-\;(8.80\times 10^{-2}\sqrt{\mathrm{BF}\%})+(9.55\times 10^{-4})\;\times \;(\mathrm{BF}\%{)}^{2}$$

The model R^2^ was 0.4447.

Furthermore, the model was used to calculate a pBMI. A paired *t*-test between the measured BMI (24.2 ± 2.6 kg/m^2^) and pBMI (24.1 ± 1.8 kg/m^2^) showed that the mean difference did not deviate from 0 (*p* = 0.358). Regressing the measured BMI on the pBMI yielded the following model with an R^2^ of 0.4905:

(2) $$\mathrm{BMI}=1.009\times e^{\mathrm{Ln}\;\mathrm{pBMI}\;}-0.158$$

The slope (β = 1.0094) was not significantly different from 1 [F (1,620) = 0.05, *p* = 0.820] with an intercept of (−0.158 kg/m^2^). Pearson’s correlation coefficient between the predicted and measured BMI was ρ = 0.7 (*p* < 0.001). The CUSUM test confirmed a linear association (*p* > 0.05) between measured BMI and pBMI.

**Figure 2:**
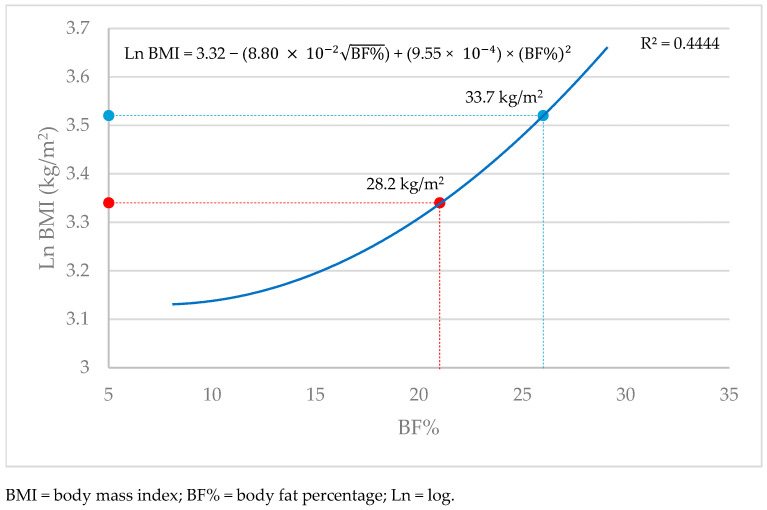
The curvilinear model illustrating the association between body fat percentage and BMI (kg/m^2^).

The substitution of BF% cut-off points for overweight (21%) and obesity (26%) resulted in BMI values of 28.2 and 33.7 kg/m^2^ to discriminate between overweight and obesity, respectively, shifting by almost three BMI units for each category. Figure 2 illustrates the curvilinear association between body fat percentage and BMI (kg/m^2^) with the new cut-off points indicated.

**Figure 3:**
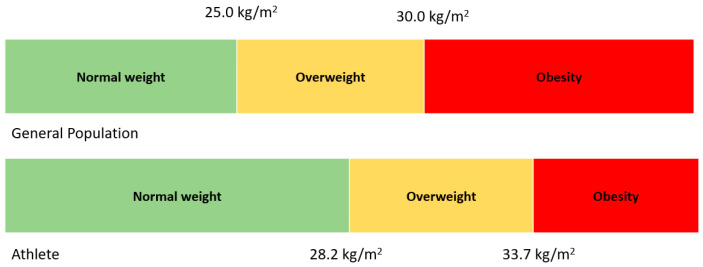
The shift in BMI among athletes relative to the general population.

Figure 3 below illustrates the shift in BMI in athletes compared to the general population.

Table 3 presents the agreement analysis between the body BF% classification [26] for normal weight, overweight and obesity and the new BMI cut-off points. According to this new BMI classification, a total of 580 (93.2%) were classified as having a normal weight and 42 (6.8%) as having overweight or obesity. The new cut-off points correctly categorized those with normal BF by 96.0%, while it captured 75.0% of those with overweight or obesity according to BF. The agreement between the two classification systems improved compared to the WHO classifications, revealing a moderate agreement (kappa = 0.522).

## 4. Discussion

The main aim of our study was to provide benchmark data about the validity of the traditional WHO BMI cut-offs of 25 and 30 kg/m^2^ for identifying overweight and obesity, respectively, in young adult athletes. In case they were found to be non-valid, the paper aimed, in a second step, to establish new BMI cut-off points that can perform more accurately when screening for overweight and obesity in this specific population.

### 4.1. Findings and Concordance with Previous Studies

Our first and main finding was that the WHO BMI cut-off points are not suitable for discriminating between normal weight, overweight and obesity, as they significantly underestimate the prevalence of individuals with normal weight, incorrectly identifying them as part of the overweight or obesity groups. As a result, the prevalence of overweight and obesity appears to have a high occurrence among young adult male athletes. In fact, in our sample, according to the WHO BMI classification, only 72% were identified as having a normal weight, whereas 24% were categorized as being with overweight and 4% with obesity. However, in reality and based on the adiposity classification (i.e., BF%), approximately 96% of our sample was of normal weight (i.e., normal fat mass), and only 3% overweight (i.e., over adiposity) and approximately 1% had obesity (i.e., excessive adiposity). This finding is perfectly in line with those of several studies that showed that a higher BMI does not necessarily imply excessive adiposity in sports environment (i.e., athletes) [20,21]. In addition, we feel that today we are in a position to clearly respond to a debated question raised over the past couple of decades regarding whether the high prevalence of overweight/obesity—according the WHO BMI classification—among athletes is fact or fiction [18].

Interestingly, our second finding was the identification of new BMI cut-off points for the identification of overweight and obesity in young male athletes. These new cut-off points (i.e., BMI = 28.2 and 33.7 kg/m^2^) in our population were found to be significantly higher than the WHO BMI cut-off points (i.e., BMI 25 and 30 kg/m^2^) [33]. Even though we are not in a position to reveal the exact reason behind this discrepancy between the BMI cut-offs for overweight and obesity in our population and those suggested by the WHO, we can, however, speculate and suggest that a different body composition at a similar BMI, such as the higher LM and lower BF in athletes with respect to their counterparts in the general population, may lead to finding over and excessive adiposity only at higher BMI levels. This result is line with previous investigations on the topic, as despite their paucity, another analysis also suggested higher cut-off points for identifying overweight or obesity in athletes [12]. For instance, a study conducted on top-level athletes noted that even a BMI of up to 32.8 kg/m^2^ may still be considered overweight for males, attributed mainly due to their high lean or fat-free mass [22].

### 4.2. Study Strengths and Limitations

Our study has particular strengths, since to the best of our knowledge, it is one of the very few analyses to test the validity of the WHO BMI cut-off points (i.e., 25 and 30 kg/m^2^) for the identification of over and excessive adiposity in a large group of athletes composed of young adult males in a “real-world” sport setting in Italy, and also to determine new BMI cut-off points which perform better in this area. Second, body composition was measured using a DXA scan, which is known to exhibit a high level of precision in the general population as well as in athletes [34,35,36]. Third, the inclusion of a large sample of athletes competing at high levels, and from several sport disciplines (i.e., soccer, basketball, volleyball, rugby, karate, tennis and others), should also be considered a strength.

However, our investigation also had some limitations. Firstly, only a sample composed of young Italian male athletes was considered, and not all types of sports were represented or sufficiently included (with only a small sub-group). Our findings therefore lack external validity since they cannot be generalized and extended to male athletes of other ethnic or age groups, or to female athletes or to all sports disciplines [37]. Secondly, we were unable to identify sport-specific BMI cut-offs due to the small sample size when the dataset was split based on sport disciplines. Thirdly, the cross-sectional design of this study should be considered another limitation since it does not facilitate the detection of the potential changes in body composition over time in this population in general as well as within each sport group [38].

### 4.3. Potential Clinical Implications and New Directions for Future Research

These findings have certain implications in the sporting environment. Firstly, and despite the very well-known and documented limitations of using BMI classification systems as an indicator of adiposity status in athletes, we invite sports committees and federations to view our results at least as preliminary evidence for the use of these new cut-off points in identifying overweight and obesity in young male athletes, rather than the traditional WHO BMI ones, which incorrectly overestimate the prevalence of these conditions (i.e., overweight and obesity). The new cut-offs appear to perform better than the old ones, especially since BMI remains an easy-to-use tool, and body composition assessment is still not always available in sporting environments. Secondly, awareness should be raised among all health professionals related to sports (i.e., sports doctors, dieticians, specialists in motor sciences, physiotherapists, etc.) involved in body assessment about recognizing these new cut-off points when screening for overweight and obesity in their athletes, and to share/discuss this advancement in knowledge with them.

However, some new directions for further research should still be considered in the future. Firstly, our findings should be replicated in larger-sample studies composed of both sexes that include the majority of sport disciplines. Such evaluations may be able to identify sport- and sex-specific BMI cut-off points for normal weight, overweight and obesity for each sport discipline in males and females, since we could not. These could take into account a newly published work on Italian athletes that reported an overall total BF% of almost 10% and 20% in male and female athletes, respectively, while splitting them by sport category. Despite a similarity in body mass, there were wide differences in BF%, which ranged between 7% and 21% in males and 20% and 27% in females [14]. Secondly, in the light of the limitations of the BMI classification system, additional investigations should also be conducted to establish other novel, simple and accurate tools that can easily determine adiposity status in athletes.

## 5. Conclusions

Our study suggests that the specific cut-off points of 25 and 30 kg/m^2^ proposed by the WHO BMI classification system are not accurate predictors of overweight and obesity, respectively, in young male athletes. However, due to the strong correlation between BF% and BMI, we developed an easy-to-use tool based on the latter in order to screen for adiposity status, which uses different cut-off points from those suggested by the WHO for the general population. Therefore, we recommend that the new cut-off points identified in our paper be applied when screening for overweight or obesity in young adult male athletes.

## Figures and Tables

**Table 1:** Anthropometric characteristics of the study participants *.

	Total Sample (n = 622)
Age (years)	25.7 ± 4.7
Weight (kg)	80.9 ± 11.8
Height (cm)	182.8 ± 9.2
BMI (kg/m^2^)	24.2 ± 2.6
Classification based on WHO BMI cut-off points:	
Normal weight	451 (72.5)
Overweight	148 (23.8)
Obesity	23 (3.7)
LM (kg)	65.8 ± 8.0
LM%	82.8 ± 3.2
BF (kg)	10.6 ± 4.1
BF%	13.1 ± 3.4
Classification based on BF% cut-off points ^§^:	
Normal weight	598 (96.1)
Overweight	19 (3.1)
Obesity	5 (0.8)

* Values are mean ± SD for continuous variables and n (%) for categorical variables. BMI = body mass index; BF = total fat mass; BF% = body fat percentage; LM = total lean mass; LM% = lean mass percentage; ^§^ BF% classification based on Gallagher classification [26].

**Table 2:** Agreement of the classification of normal weight and of overweight or obesity between the WHO BMI and BF% classification systems.

		BF% Classification *			
	Total (n = 622)	Normal Weight (n = 598)	Overweight or Obesity (n = 24)	Significance	Kappa
	n (%)				
WHO BMI categories				X^2^ = 51.575; *p* < 0.001	K = 0.169; *p* < 0.001
Normal weight	451 (72.5)	449 (75.1)	2 (8.3)		
Overweight or obesity	171 (27.5)	149 (24.9)	22 (91.7)		

BMI = body mass index; BF% = body fat percentage; * BF% classification based on Gallagher classification [26].

**Table 3:** Agreement of the classification of normal weight and of overweight or obesity between the new BMI and BF% classification systems.

		BF% Classification *			
	Total (n = 622)	Normal Weight (n = 598)	Overweight or Obesity (n = 24)	Significance	Kappa
	n (%)				
New BMI categories				X^2^ = 184.662; *p* < 0.001	K = 0.522; *p* < 0.001
Normal weight	580 (93.2)	574 (96.0)	6 (25.0)		
Overweight or obesity	42 (6.8)	24 (4.0)	18 (75.0)		

BMI = body mass index; BF% = body fat percentage; * BF% classification based on Gallagher classification [26].

## Data Availability

The original contributions presented in this study are included in the article. Further inquiries can be directed to the corresponding author.
